# Minimum joint space width and tibial cartilage morphology in the knees of healthy individuals: A cross-sectional study

**DOI:** 10.1186/1471-2474-9-119

**Published:** 2008-09-08

**Authors:** Karen A Beattie, Jeffrey Duryea, Margaret Pui, John O'Neill, Pauline Boulos, Colin E Webber, Felix Eckstein, Jonathan D Adachi

**Affiliations:** 1 Dept. of Medicine, McMaster University, Hamilton, ON, Canada; 2 Dept. of Radiology, Brigham & Women's Hospital, Boston, MA, USA; 3 Dept. of Radiology, Scarborough General Hospital, Scarborough, ON, Canada; 4 Dept. of Radiology, St. Joseph's Healthcare, Hamilton, ON, Canada; 5 Hamilton Health Sciences, Hamilton, ON, Canada; 6 Paracelsus Private Medical University, Salzburg, Austria

## Abstract

**Background:**

The clinical use of minimum joint space width (mJSW) and cartilage volume and thickness has been limited to the longitudinal measurement of disease progression (i.e. change over time) rather than the diagnosis of OA in which values are compared to a standard. This is primarily due to lack of establishment of normative values of joint space width and cartilage morphometry as has been done with bone density values in diagnosing osteoporosis. Thus, the purpose of this pilot study is to estimate reference values of medial joint space width and cartilage morphometry in healthy individuals of all ages using standard radiography and peripheral magnetic resonance imaging.

**Design:**

For this cross-sectional study, healthy volunteers underwent a fixed-flexion knee X-ray and a peripheral MR (pMR) scan of the same knee using a 1T machine (ONI OrthOne™, Wilmington, MA). Radiographs were digitized and analyzed for medial mJSW using an automated algorithm. Only knees scoring ≤1 on the Kellgren-Lawrence scale (no radiographic evidence of knee OA) were included in the analyses. All 3D SPGRE fat-sat sagittal pMR scans were analyzed for medial tibial cartilage morphometry using a proprietary software program (Chondrometrics GmbH).

**Results:**

Of 119 healthy participants, 73 were female and 47 were male; mean (SD) age 38.2 (13.2) years, mean BMI 25.0 (4.4) kg/m^2^. Minimum JSW values were calculated for each sex and decade of life. Analyses revealed mJSW did not significantly decrease with increasing decade (p > 0.05) in either sex. Females had a mean (SD) medial mJSW of 4.8 (0.7) mm compared to males with corresponding larger value of 5.7 (0.8) mm. Cartilage morphometry results showed similar trends with mean (SD) tibial cartilage volume and thickness in females of 1.50 (0.19) μL/mm^2 ^and 1.45 (0.19) mm, respectively, and 1.77 (0.24) μL/mm^2 ^and 1.71 (0.24) mm, respectively, in males.

**Conclusion:**

These data suggest that medial mJSW values do not decrease with aging in healthy individuals but remain fairly constant throughout the lifespan with "healthy" values of 4.8 mm for females and 5.7 mm for males. Similar trends were seen for cartilage morphology. Results suggest there may be no need to differentiate a t-score and a z-score in OA diagnosis because cartilage thickness and JSW remain constant throughout life in the absence of OA.

## Background

Osteoarthritis (OA) is the most common joint disorder and is responsible for substantial economic, social and psychological costs. While the prevalence of OA is variable based on how the disease is defined, it has been said that the majority of individuals over the age of 65 years living in the western world demonstrate radiographic evidence of disease [1-3]. Conventional radiography has been, and continues to be, the primary imaging modality used in the evaluation of OA, both in terms of diagnosis and monitoring of disease progression. In the knee joint, osteoarthritic features visible on radiographs include joint space narrowing, osteophytosis, subchondral osteosclerosis and subchondral cysts.

The measurement of the separation between the distal femur and the proximal tibia, joint space width (JSW), has become the standard tool for the assessment of knee OA progression [4]. Both the fluoroscopic and non-fluoroscopic acquisition of radiographs have allowed for the evaluation of JSW revealing good precision of measurement [5-9]. Standard X-rays acquired using the non-fluoroscopic fixed-flexion technique can be as reproducible as fluoroscopic techniques (root-mean-square standard deviation = 0.1 mm) with the added advantages of lower costs and considerably less radiation dose [9,10], although they have been shown to be less sensitive to change in knee OA patients than the Lyon-Schuss fluoroscopic technique [11]. Also, it has been shown that the reproducibility of measurement of minimum joint space width (mJSW) is better when using an automated computer algorithm as compared to manual methods such as a hand-held lens [9,10,12,13].

Cartilage thinning is the signature feature of knee OA and JSW measurements should be an indirect measure of the articular cartilage thickness in the joint. The emergence of magnetic resonance imaging as an important tool in the visualization of articular cartilage together with dedicated image analysis software permits one to quantify cartilage volume and thickness directly [14-19].

Despite the advances in techniques used to evaluate joint space width and cartilage volume and thickness, their use in clinical studies has been limited to the longitudinal measurement of disease progression (i.e. change over time) rather than the diagnosis of OA in which values are compared to a standard as has been done in the diagnosis of osteoporosis where there are age dependent normal values of bone mineral density [20-23]. The potential for age dependent normal values of mJSW and cartilage morphometry may also be the case, as investigated here. In addition, since joint space width measures are thought to be a surrogate measure of cartilage thickness, one might hypothesize that these variables are correlated with one another. Although one study investigating the relationship between these variables has been previously conducted in osteoarthritic individuals, the strength of this relationship has not been explored in healthy individuals. A healthy reference is needed in order to determine how these relationships compare or change under osteoarthritic conditions. Thus, the purpose of this pilot study was to estimate reference values of medial minimum joint space width and cartilage morphometry in healthy males and females between the ages of 20 and 69 years using standard radiography and peripheral magnetic resonance imaging (pMRI) and to further investigate the correlation between medial tibial cartilage thickness and medial minimum joint space width.

## Methods

Healthy volunteers between 20 and 69 years of age with no known bone or joint disease were recruited to undergo a knee X-ray and a peripheral magnetic resonance scan of the same knee via locally posted advertisements and word of mouth. Individuals were asked to respond to the advertisements by telephone and were posed a number of screening questions to ensure that study inclusion and exclusion criteria were met. Those excluded from participating (N = 8) were those who a) stated they were currently experiencing knee pain, b) stated they had been previously diagnosed with a bone or joint disease (i.e. rheumatoid arthritis, osteoporosis), or c) had previously sustained a knee injury and/or had undergone any knee surgery (i.e. arthroscopy, menisectomy, etc.). Only those with radiographically normal X-rays were included in the analyses. All participants were required to sign a consent form that had been approved by the Research Ethics Board at St. Joseph's Healthcare. In addition to completing the study consent form, individuals were required to complete a questionnaire which asked questions pertaining to his/her medical history, medications and exercise activity.

### Plain X-ray

Study participants underwent a single knee X-ray of the non-dominant knee acquired in the fixed-flexion position [9,10]. In this position, participants are required to stand, with their weight distributed equally between their legs, on a piece of cardboard such that both great toes are touching the vertical X-ray table and feet are externally rotated by approximately 10°. Both feet are traced onto cardboard should the foot map be needed for use in successive X-rays. Facing the vertical X-ray table and holding the sides for balance and support, subjects are asked to bend their knees slightly such that both their patellas and thighs are pressed tightly against the table. In doing so, the position of the femur and tibia are fixed, and thus, so is the degree of knee flexion. The posteroanterior X-ray beam is directed parallel to the tibial plateau at a 10° caudal beam alignment.

Radiographs were graded independently by two musculoskeletal radiologists according to the Kellgren-Lawrence (K-L) scoring system [24]. This grade was used to confirm or refute the presence of knee OA. Those assigned a grade of 0 or 1 on the scale were included. Individuals whose X-rays scored ≥ 2 on the K-L scale were excluded from the analyses because they had evidence of knee OA.

**Figure 1:**
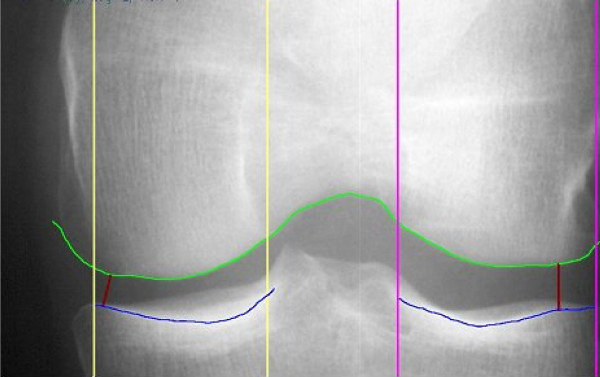
A digitized radiograph analyzed for minimum joint space width using an automated computer algorithm (medial compartment on left).

X-ray films were subsequently digitized using a Sierra plus™ digitizer (Vidar Systems Corporation, Herndon, VA, USA) at an isotropic pitch of 84.7 μm and a 12 bit grey scale resolution. The digitized images were further analyzed for mJSW in the medial compartment of the knee using a automated computer algorithm, details of which have been described previously [12]. The reproducibility of this analysis technique has been shown to be very good (RMSSD = 0.15 mm; CV = 3.31% in healthy individuals). An analyzed radiograph is depicted in Figure 1. This program delineates the bony margins of the femoral condyles and the tibial plateau. In approximately 3% of radiographs analyzed for mJSW, user intervention was required to slightly alter the delineations drawn by the computer algorithm.

### pMRI

Peripheral MR scans were acquired using a 1.0 Tesla peripheral MRI (pMRI) system (OrthOne™, ONI Inc., Wilmington, MA, USA). Subjects were seated in the scanning chair with their knee fully extended and centred within the iso-centre of the 180 mm removable quadrature volume transmit-receive coil. Padding was placed around the knee, thigh and leg to limit the potential for movement inside the magnet. All study participants were positioned and scanned by the same technologist.

**Figure 2:**
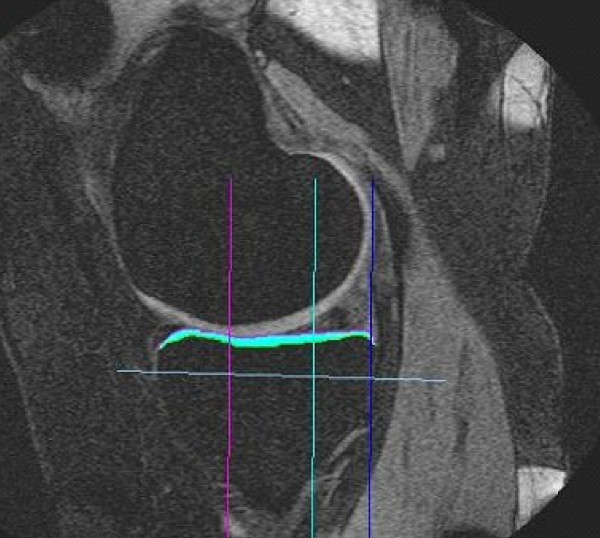
Medial tibial cartilage segmented from a single sagittal slice of an MR image acquired from a healthy knee.

Sagittal gradient-echo and axial fast spin-echo localizer scans were performed (total scan time 2–3 minutes). Following this, a fat-saturated spoiled gradient recalled acquisition in the steady state (SPGR) was performed in the sagittal plane using the following parameters: TR 60 ms; TE 12.4 ms (or minimum); flip angle 40°; bandwidth 30 kHz; matrix 512 × 256 (frequency × phase); 1 excitation; field of view 150 mm; slice thickness 1.5 mm; 56 to 64 partitions depending on patient size; scan time 15–16 minutes. Images were transferred to an independent workstation where they were saved in DICOM format. Upon completing the acquisition of all images, one trained technician conducted analyses to quantify the cartilage morphology of the medial tibia (MT) using a reproducible, validated proprietary segmentation software program (Chondrometrics GmbH, Ainring, Germany) [25-29]. Cartilage segmentation was conducted on a slice-by-slice basis (number of slices was dependent upon patient size) by manual tracing the bone-cartilage interface and the cartilage surface of the entire MT [25,26]. This segmentation algorithm has previously been validated in the pMRI [26]. After segmenting all MT plates, images were reviewed a second time for the purposes of quality assurance and adjustments in segmentation were made if deemed necessary. Total volume of MT cartilage (VC), cartilage volume normalized to medial tibial bone size (VCtAB), and cartilage thickness over the total area of medial tibial subchondral bone (ThCtAB) were computed [25,30]. An example of a single slice of segmented MT cartilage is shown in Figure 2.

## Results

In total, 119 healthy individuals with no history of knee pain or injury and without a bone or joint disease agreed to participate in the X-ray portion of the study. Of these, 73 were female and 46 were male and all but 3 were Caucasian. Demographic data are presented in Table 1.

**Table 1:** Study population demographic statistics

*N (females)*	*119 (73)*
Age (SD) (yrs)	38.2 (13.2)
BMI (SD) (kg/m^2^)	25.0 (4.4)
K-L grades (N):	
0	80
1	39

K-L grading of X-rays revealed that, of the women, 49 had radiographic scores of 0 while the remaining 24 had scores of 1. Grading for males revealed that 31 had K-L scores of 0 and 15 had a score of 1. Of the 119 individuals who participated in the X-ray portion of the study, 86 also underwent a pMRI scan of the same knee, 50 of whom were female and 36 of whom were male. It should be noted that the mean age and BMI of individuals who received pMR scans was very similar to the entire study population (mean age 38.3 yrs, mean BMI 25.3 kg/m^2^) suggesting that results may be generalizable to the overall study group.

Subjects were subdivided according to decade of life for the analysis of mJSW, thus treating age as a categorical variable. The purpose of doing so was to determine if there was an identifiable decade where initial changes (i.e. decreases) in mJSW could be detected. In addition, other groups performed analyses by age decades thus allowing comparisons to be made between studies [31-33]. The mean (SD), range, minimum and maximum mJSW data for each of these age groups were calculated and are presented in Table 2.

**Table 2:** mJSW data per sex and decade in healthy individuals

	**Age Group (yrs)**	**N**	**K-L Grade 0 (%)**	**Mean (mm)**	**SD (mm)**
**Females**	20 – 29	22	91	5.06	.56
	30 – 39	15	80	4.62	.66
	40 – 49	14	43	4.84	.69
	50 – 59	17	47	4.75	.93
	60 – 69	5	60	4.61	.44
**Males**	20 – 29	18	89	5.55	.51
	30 – 39	13	46	5.76	.71
	40 – 49	7	86	5.35	1.08
	50 – 59	6	33	5.43	.71
	60 – 69	2	50	5.41	.56

The descriptive statistics do not appear to show any differences in mean mJSW values between decades in either males or females. In fact, these cross-sectional data suggest that a mean (SD) "normal" value of mJSW for healthy women is 4.8 (0.7) mm while in healthy men this value is larger at 5.7 (0.8) mm. This was supported by results from an ANOVA analysis in which no significant differences in mJSW were found between age groups in either men or women even after considering BMI as a covariate (p > 0.05). The only significant difference identified was that between genders where an ANOVA analysis performed with BMI, age and gender as covariates revealed that males have significantly larger mJSW values than females (p < 0.05).

Analyses were repeated with age as a continuous rather than a categorical variable with age, BMI and gender considered independent variables. While age and BMI were not predictive of mJSW, gender was again found to be significant with healthy males having significantly larger mJSW values compared to healthy females (β regression coefficient = 0.84, p < 0.001).

Cartilage analyses were also conducted in an attempt to determine "normal" cartilage volume and thickness values in healthy males and females of different age groups. These data are presented in Table 3. The mean (SD) medial tibial cartilage volume normalized to the area of subchondral bone was 1.50 (0.19) μL/mm^2 ^in females while in males it was 1.77 (0.24) μL/mm^2^. Corresponding mean values for cartilage thickness over the entire bone surface were 1.45 (0.19) mm and 1.71 (0.24) mm in females and males, respectively.

**Table 3:** Medial tibial cartilage data per sex and decade in healthy individuals

	**Age Group (yrs)**	**N**	**Mean (SD)** **VCtAB (μL/mm^2^)**	**Mean (SD)** **ThCtAB (mm)**
**Females**	20 – 29	13	1.52 (0.15)	1.45 (0.14)
	30 – 39	11	1.60 (0.25)	1.54 (0.24)
	40 – 49	11	1.49 (0.16)	1.44 (0.15)
	50 – 59	11	1.42 (0.20)	1.38 (0.19)
	60 – 69	4	1.40 (0.12)	1.35 (0.11)
**Males**	20 – 29	13	1.78 (0.28)	1.71 (0.27)
	30 – 39	11	1.78 (0.26)	1.73 (0.28)
	40 – 49	3	1.67 (0.23)	1.61 (0.24)
	50 – 59	6	1.82 (0.18)	1.75 (0.13)
	60 – 69	2	1.85 (0.19)	1.80 (0.14)

Just as with mJSW values, medial tibial cartilage volume and thickness data did not appear to differ significantly between age groups for healthy males or females. This observation was confirmed by ANOVA analyses which revealed no significant differences in VCtAB or ThCtAB values between different age groups (p > 0.05). However, significant differences were found between genders with males consistently having thicker cartilage than their female counterparts.

While investigating the relationship between medial tibial cartilage morphometry and age as a continuous variable in males, the relationship did not change from that considering age as a categorical variable. Regression analyses with BMI as a covariate showed that medial tibial cartilage volume, normalized to bone area, and thickness did not decrease significantly with age (p > 0.05). However, this was not the case for females. Both cartilage volume and thickness in the medial tibia appeared to decrease with ageing showing standardized regression coefficients of -0.41 (p = 0.008) and -0.37 (p = 0.015), respectively.

Analyses were also conducted to investigate the relationship between medial tibial cartilage morphometry and medial mJSW, since mJSW is considered to be a surrogate measure of cartilage thickness. Between cartilage volume (VC) and mJSW, correlation analyses revealed a correlation coefficient of 0.67 while the correlation between VCtAB and mJSW was 0.69 and the correlation between ThCtAB and mJSW was also 0.69. These results suggest that approximately 47% of the variation in mJSW can be explained by the variation in cartilage thickness of the medial tibia.

## Discussion

The primary purpose of establishing normal values of mJSW in a healthy population of males and females is to provide age-specific references to which osteoarthritic values can be compared. In addition, it is important to determine if mJSW values appear to decrease with age in a healthy population or if, indeed, this is characteristic of only those affected by knee OA. We also investigated the correlation between mJSW and medial tibial cartilage morphometry in this healthy population. Results of this pilot study appear to suggest that mJSW values are not significantly different between younger and older individuals without radiographic evidence of OA as shown by mean (SD) values of 4.8 (0.7) mm and 5.7 (0.8) mm in females and males, respectively. Individuals included in these analyses were those with K-L grades of 0 and 1 as was the case in a recent study by Conrozier et al. [5]. While it may be argued that a K-L grade of 1 may correspond to early OA, the definition of this categorization states the doubtful presence of osteophyte without regard for joint space narrowing. This would support the notion that cartilage thickness measurements would not be affected by the inclusion of those with K-L grade 1. To verify this, additional analyses including only those with K-L grades of 0 (grade 1 excluded) were performed and results did not differ significantly from those which included both K-L grades 0 and 1.

Results from this study suggest that there is no identifiable decade of life when one might expect joint space width to narrow. When considered as a continuous variable, age was not found to be significantly related to mJSW in either males or females again supporting the notion that joint space narrowing may not simply be a consequence of aging. To confirm this, however, we recognize that a longitudinal study collecting data over decades would be required and therefore was not feasible at this point in time.

Despite the fact that there are few studies which are longitudinal in nature, there are a few cross-sectional studies which have investigated the relationship between JSW measurements and age with methodologies slightly different than the ones used in the present study. For example, a study of healthy young adults 16–22 years of age reported mean (SD) medial mJSW values of 4.74 (0.94) and 5.65 (0.93) in females and males respectively, results much like those of similar aged participants in this study [34]. Also like our study, JSW values in 125 healthy individuals between 40 and 75 years of age were not found to decrease with increasing decade of life with mean values ranging from 4.6 – 5.0 mm in females and 5.0 – 5.5 mm in males [31]. In both of these studies, males were generally found to have larger JSWs compared to females, results which are also consistent with those reported here [31,34]. In contrast, studies conducted by Dacre et al. and Sargon et al. showed that JSW decreased with increasing age group, although both of these studies were cross sectional in nature and methodological differences existed including X-ray acquisition technique (weight bearing vs. non-weight bearing), joint space analysis (manual vs. automated, joint space area (mm^2^) vs. mJSW [33]) and the symptomatic nature of patients [32,33]. While our results and those of other cross-sectional studies of healthy individuals suggest mJSW values remain constant, other results suggesting the opposite justify the need for large-scale, cross-sectional and longitudinal population-based data of healthy individuals acquired using the most reproducible techniques [22,35].

Although there is a paucity of radiographic data from healthy individuals conducted over time, a study conducted by Conrozier et al. examined longitudinal changes in mJSW in individuals reporting chronic knee pain (>3 months) but lacking radiographic evidence of knee OA (K-L grade ≤ 1), as was considered in the present study. These authors reported a mean (SD) annual rate of joint space narrowing of 0.05 (0.22) mm [5]. However, the symptomatic nature of the participants may be indicative of cartilage lesions that may not be radiographically detectable, as reported by Ding et al. in patients with K-L grade 1, thereby questioning the status of this sample as a "healthy" population [36]. In addition, this study did not report whether this change was statistically significant from baseline to one-year follow-up. In fact, such small changes in joint space width are often within the range of reproducibility error of measurement [5].

Other studies of medial JSW values in healthy individuals have reported average values for the entire populations under investigation but have not analyzed these measurements as they varied with age or sex [32,37]. Dacre et al., for instance, reported the mean medial JSW acquired from non-weight-bearing radiographs to be 5.73 (0.15) mm in females and 7.03 (0.12) mm in males, results which are 17% and 19% larger than those of the present study, respectively [33]. However, joint space width values acquired from non weight-bearing X-rays may be larger than those acquired from weight-bearing ones, suggesting that these results may, indeed, be consistent with those of the present study [38].

Given that mJSW is a surrogate measure of cartilage thickness, one would hypothesize that these variables would be correlated with one another. However, it is widely understood that joint space width measurements reflect only a thickness measure at one specified location in the joint and may include tissues such as menisci and synovial fluid, findings that are supported by previously published studies [39-44]. Our results revealed that the variance in medial tibial cartilage thickness, normalized to bone area, can explain less than half of the variation in medial mJSW. It must be noted here, however, that medial femoral cartilage thickness was not analyzed in this study population. This variable would certainly also account for some of the variation in joint space, although cartilage thickness in one plate is not highly correlated with cartilage in another plate [45]. Analyses previously conducted in an osteoarthritic population where both medial femoral and tibial cartilage were examined suggested the variation in cartilage thickness accounted for 54% of mJSW [46].

The issue of whether sex and age are significantly related to cartilage volume and thickness has been the subject of many studies [47-51]. Mixed results have been reported with respect to gender differences in cartilage volume and thickness, although our results revealed that males have significantly larger mean tibial cartilage volume and thickness just as was the case with mJSW. Similarly, studies by Faber et al. and Cicuttini et al. reported significantly larger mean cartilage volume values in healthy males compared to healthy females [49,51-53]. While Cicuttini et al. also reported significantly larger medial tibial cartilage thickness values in males of the same population, although thickness was not assessed directly but calculated as volume per unit area, Faber et al. did not find such significant differences in thickness between genders [49,51,53]. For instance, in our study, men had 18% more medial tibial cartilage thickness compared to women while Faber demonstrated that men had 13.3% thicker cartilage than women, although this difference was not statistically significant. Discrepancies in results from these studies likely exist because of differences in sample populations (i.e. age, definition of "healthy") and the relatively small sample size.

In the current study, in contrast to results investigating age as a categorical variable, increasing age (as a continuous variable) was found to be associated with less cartilage volume normalized to total bone area (β = -0.41) and thickness (β = -0.37) in females after adjusting for BMI. Such age-related differences in medial tibial cartilage volume and thickness were not observed in males. While this may be related to the relatively small number of healthy males over 50 years of age in our study sample, it is also possible that inconsistencies between males and females may be related to hormonal changes which occur during menopause of which there are no comparable changes that occur in men. This is similar to the BMD findings in osteoporosis [54,55]. Other cross-sectional studies which have investigated the relationship between age and cartilage volume and thickness have shown inconsistent results with one reporting a significant decrease in medial cartilage thickness, but not volume, with age [56], while another reports no significant changes in tibial cartilage thickness with age [22]. However, one should be cautious about the interpretation of these results since these data are cross-sectional in nature and do not reflect changes in a single person over time but comparisons between different individuals.

Three studies have reported longitudinal changes in cartilage volume for healthy individuals and have shown that cartilage volume does, indeed, decrease with aging [54,57,58]. In healthy males (N = 28, mean age 52 years), the mean annual reduction in tibial cartilage volume was found to be 2.8% (95% CI = 0.2% to 5.5%) [54]. In healthy postmenopausal females, the average annual decrease in total tibial cartilage volume was similar at 2.4% (3.2%) [58]. What is notable in these two studies is the mean age of subjects being investigated was over 50 years. To this point, there is only one study investigating longitudinal changes in a population including younger adults. Ding et al. demonstrated a significant association between age and loss of cartilage volume by approximately 1.5 – 4.2% per annum in individuals between the ages of 26 and 60 years, with a higher rate of loss in females as compared to males [47]. However, despite these seemingly age-related declines, it is still plausible that these values lie within what may be considered to be a "normal" or "healthy" range.

It is important to recognize that there are a number of methodological limitations to this study including the small sample sizes, particularly in some age groups, the cross-sectional nature of the data and the lack of medial femoral cartilage analyses. Despite these limitations, results suggest that mJSW values do not decrease with increasing age group in males or females between the ages of 20 and 69 years. This information may be helpful in defining radiographic joint space width references for comparison with those suspected of having knee OA. These results also suggest that there is no defined decade at which point joint space width decreases. Cartilage volume and thickness did not decrease with increasing age in males as was the case with mJSW. However, the observation that cartilage volume and thickness decreased with ageing in females may support the role of estrogen in cartilage physiology, although the exact mechanism remains unknown. It is also possible that tissue other than medial tibial cartilage may play a more significant role in joint space narrowing than in males, although this has not yet been shown.

## Conclusion

The results of this cross-sectional pilot study investigating the knees of healthy individuals suggest that mJSW measures from plain radiographs remain relatively constant through the third to seventh decades of life. The lack of significant declines associated with ageing also suggests that mJSW values may be helpful for comparisons with those suspected of having knee OA. In males, these results are supported by cartilage volume and thickness data which also remain fairly constant throughout the middle ages. However, decreasing values of cartilage thickness and volume in females over the ages suggest that discrepancies with mJSW results may be due to tissue other than medial tibial cartilage or another mechanism yet to be fully elucidated.

## Competing interests

Dr. Felix Eckstein is the founder and CEO of Chondrometrics GmbH. This is the company responsible for providing the cartilage segmentation software that was used in this study. There are no other conflicts of interest to mention.

## Authors' contributions

KB, PB, JDA and CW conceived of the study, and participated in its design and coordination and helped to draft the manuscript. JD assisted in the automated analyses of the radiographs while MP and JO read and scored radiographs using the Kellgren-Lawrence scale while FE assisted in the cartilage morphometry analyses of the pMR images. All authors read and approved the final manuscript.

## Pre-publication history

The pre-publication history for this paper can be accessed here:


